# Comparison of Methods for Feature Selection in Clustering of High-Dimensional RNA-Sequencing Data to Identify Cancer Subtypes

**DOI:** 10.3389/fgene.2021.632620

**Published:** 2021-02-24

**Authors:** David Källberg, Linda Vidman, Patrik Rydén

**Affiliations:** ^1^Department of Statistics, USBE, Umeå University, Umeå, Sweden; ^2^Department of Mathematics and Mathematical Statistics, Umeå University, Umeå, Sweden; ^3^Department of Radiation Sciences, Oncology, Umeå University, Umeå, Sweden

**Keywords:** feature selection, gene selection, RNA-seq, cancer subtypes, high-dimensional

## Abstract

Cancer subtype identification is important to facilitate cancer diagnosis and select effective treatments. Clustering of cancer patients based on high-dimensional RNA-sequencing data can be used to detect novel subtypes, but only a subset of the features (e.g., genes) contains information related to the cancer subtype. Therefore, it is reasonable to assume that the clustering should be based on a set of carefully selected features rather than all features. Several feature selection methods have been proposed, but how and when to use these methods are still poorly understood. Thirteen feature selection methods were evaluated on four human cancer data sets, all with known subtypes (gold standards), which were only used for evaluation. The methods were characterized by considering mean expression and standard deviation (SD) of the selected genes, the overlap with other methods and their clustering performance, obtained comparing the clustering result with the gold standard using the adjusted Rand index (ARI). The results were compared to a supervised approach as a positive control and two negative controls in which either a random selection of genes or all genes were included. For all data sets, the best feature selection approach outperformed the negative control and for two data sets the gain was substantial with ARI increasing from (−0.01, 0.39) to (0.66, 0.72), respectively. No feature selection method completely outperformed the others but using the dip-rest statistic to select 1000 genes was overall a good choice. The commonly used approach, where genes with the highest SDs are selected, did not perform well in our study.

## Introduction

The human genome consists of around 21,000 protein coding genes (Pertea et al., 2018). By analyzing genes using high-throughput technologies (e.g., sequence and microarray technologies), researchers get access to huge amount of data that can be of relevance for prognosis of a disease (classification), identification of novel disease subtypes (cluster analysis) and detection of differentially expressed genes. Aside from the fact that the number of genes often far exceeds the number of samples, most features (i.e., genes) contain no information related to the trait of interest. Whether the aim is to distinguish between different tumor stages or identifying new disease subtypes, the identification of discriminating features is key.

Diseases like cancer arise by various causes and there is reason to believe that today’s cancer diseases can be divided further into several subtypes, which potentially should be treated differently. Cluster analysis applied on gene expression data from samples (e.g., tumor samples or blood samples) taken from cancer patients has successfully been used to detect novel cancer subtypes (Eisen et al., 1998; Sotiriou et al., 2003; Lapointe et al., 2004; Bertucci et al., 2005; Fujikado et al., 2006; Ren et al., 2016). However, the problem of detecting new subtypes is challenging since most of the genes’ expressions are not affected by disease subtype and some genes are influenced by other factors such as gender, age, diet, presence of infections and previous treatments. Ideally, a cluster analysis aimed at detecting novel disease subtypes should only utilize genes that are informative for the task, i.e., genes that have their expression mainly governed by which disease subtype the patient has. Hence, it is of interest to apply some sort of gene selection procedure prior to the cluster analysis. This task would be relatively easy if it was known which subtypes (i.e., labels) the patients have, but for unsupervised classification problems, the labels are unknown making gene selection a true challenge. When the labels are unknown, statistical tests such as *t*-tests, Wilcoxon rank sum tests or one-way ANOVA cannot be used to identify differentially expressed genes. Instead, other data characteristics need to be considered. For example, a common approach to discover subgroups in high-dimensional genomic data is to apply clustering on a subset of features that are selected based on their standard deviation (SD) across samples (Bentink et al., 2012; Kim et al., 2020; Shen et al., 2020). Thus, the SD is used as a score that measures how informative a gene is for the underlying subgroups. Here we also consider a set of alternative scores for selecting informative genes, i.e., genes affected by the subtype. Aside SD, other examples within the category of variability scores include, e.g., the interquartile range (IQR) and measures based on entropy (Liu et al., 2005; Seal et al., 2016). If instead it is assumed that informative genes are likely to be expressed at a relatively high level it makes sense to select highly expressed genes. Another class of measures is based on quantifying the extent to which the gene expression distribution can be described by two or more relatively distinct peaks, or modes, which represent different subtypes. In the simplest case, we assume that the tumor samples can be divided into two subtypes. Given that this assumption is true, the gene expression of an informative gene may have a bimodal distribution. By ranking genes according to some bimodality measure and including only the top scoring genes (i.e., the genes with the highest bimodality measures), it is possible to remove uninformative and redundant genes before performing clustering. Several gene selection procedures based on bimodality have been proposed (Moody et al., 2019), including the bimodality index (BI; Wang et al., 2009), the bimodality coefficient (BC; SAS Institute, 1990) and various variants of the variance reduction score (VRS; Bezdek, 1981; Hellwig et al., 2010). A more general approach is to search for genes with an apparent multimodal distribution. The dip-test suggested by Hartigan and Hartigan (1985) addresses this problem.

It may be argued that genes that are involved in the same biological processes should have similar expression profiles across samples (Wang et al., 2014). Under the assumption that a fair number of genes are affected by the disease subtype, it is natural to search for a large set of genes that are highly correlated. In the established taxonomy for feature selection approaches, the methods studied here are *filtering* methods, other important classes are wrapper, embedded, and hybrid methods thereof (Ang et al., 2016).

It is evident that fundamentally different selection procedures will identify different sets of genes. Moreover, several of the approaches are likely to include not only informative genes but also genes affected by other factors and genes that have general inclusion properties (e.g., genes with highly variable gene-expressions). In the worst-case scenario, a gene selection may fail to identify genes associated with the subtype partition of interest. This risk is particularly relevant if the influence of the disease subtype is weak compared to other factors. Hence, gene selection can have a negative influence on the clustering performance.

Multiple studies have compared feature selection methods where the ultimate goal is to classify patients according to some disease status. Arun Kumar et al. (2017) compared feature selection algorithms based on execution time, number of selected features and classification accuracy in two microarray gene expression data sets. Abusamra (2013) compared eight feature selection methods on two publicly available microarray gene expression data sets of glioma and found that no single method outperformed the others. Cilia et al. (2019) concluded that the feature selection process plays a key role in disease classification and that a reduced feature set significantly improved classification, but no selection method had a superior performance in all data sets. Much fewer studies have compared feature selection methods where the objective is detection of novel subgroups using clustering (Freyhult et al., 2010).

Here we focus on evaluating and comparing means of selecting informative genes in high-dimensional RNA-seq data from human cancers before performing cluster analysis for identification of subtypes. The study is extensive and evaluates 13 gene selection procedures on four human cancer tumor types, each with two known subtypes. The approaches are compared to two negative controls (including all genes or a set of randomly selected genes) and a positive control (genes selected using label information). We study the performance of the methods, properties of the selected genes and overlap between sets of selected genes. We also investigate how the performance changes when the relative distribution of the subtypes is altered.

## Materials and Methods

### Data

Experimental RNA-sequencing raw count data from the TCGA-database were obtained through Broad institute GDAC Firehose^1^. Four different cancer types with known subgroups were used in the analyses: breast (BRCA), kidney (KIRP), stomach (STAD), and brain (LGG) cancer. In all evaluations we treated the defined cancer subtypes as gold standard partitions, although there exist several ways of grouping the data.

The *Brain data* (denoted LGG by TCGA) consists of data from 226 tumor samples from patients with lower grade glioma, where 85 patients had the IDH mutation and 1p/19q co-deletion (IDHmut-codel) while the remaining 141 patients had the IDH mutation without the 1p/19q co-deletion (IDHMut-NOcodel)(Brat et al., 2015). The *Breast data* (BRCA by TCGA) consists of data from 929 tumor samples from patients with breast invasive carcinoma (BRCA), where 216 patients had negative Estrogen Receptor status (ER−) while the remaining 713 patients had positive ER status (ER+). The *Kidney data* (KIRP by TCGA) includes data from tumor samples from 150 patients with kidney renal papillary cell carcinoma (KIRP), where 73 patients were histologically determined as subtype 1 and the remaining 77 samples were determined as subtype 2 (The Cancer Genome Atlas Research Network, 2016). The *Stomach data* was obtained from tumors in 178 patients with stomach adenocarcinoma (STAD), where 55 patients had microsatellite instability (MSI) tumors and the remaining 123 patients had tumors with chromosomal instability (CIN) (Cancer Genome Atlas Research Network, 2014).

### Clustering of Samples

Raw gene level count data were obtained from the TCGA-database, i.e., an integer value was observed for each sample and gene. First, the raw data were pre-processed, including initial filtration, between sample normalization and applying a variance stabilizing transformation, see section “Pre-processing” for further details. A variety of gene selection approaches were applied to the pre-processed data, see section “Selection of Informative Genes”. Hierarchical clustering using Ward’s linkage and the Euclidean distance was performed on the selected genes. In addition, *k*-means (*k* = 2) clustering (Hartigan and Wong, 1979) and hierarchical clustering using Ward’s linkage and a correlation-based distance (i.e., 1-| ρ|, where ρ is the Spearmans correlation coefficient) were performed in some selected cases. The two major groups identified by the clustering algorithm defined a binary sample partition that was compared to our gold standard partition (i.e., the partition defined by the considered subgroups), see section “Evaluations”.

### Simulation Study

Prior to analyzing the cancer data, a small simulation study was conducted to understand if inclusion of non-informative features (here defined as features with identically distributed feature values) has a negative effect on the clustering performance. Data from 100 samples (50 labeled A and 50 labeled B) with 10,000 features were simulated. Here, 100 features were informative such that the A-values were simulated from a normal distribution with mean 0 and variance 1 [i.e., *N*(0,1)] and the B-values were simulated from *N*(1,1). All the non-informative values were simulated from *N*(0,1). Hierarchical clustering using Ward’s linkage and the Euclidean distance was performed on: all features, only the 100 informative features and the *k* features with the highest SD, *k* = 100, 200, …, 10,000. The simulations were repeated 40 times. For each clustering, the performance was measured using the adjusted Rand index (ARI) (Hubert and Arabie, 1985), where the clustering result was compared to the AB-partition.

### Pre-processing

All four data sets originally contained gene expression for 20,531 genes. As a first step in finding informative genes, we excluded genes expressed at low levels. A score was constructed for each gene by counting the number of samples with expression values below the 25th gene percentile (i.e., the expression value below which 25% of the genes in a sample can be found). The 25% of the genes with highest score were filtered out. Next, the R-package *DESeq2* (Love et al., 2014) was used for between sample normalization using the standard settings. Finally, the normalized data was transformed using a variance-stabilizing transform (VST), which conceptually takes a given variance-mean relation σ^2^ = *var*(*x*) = *h*(μ) and transforms the data according to

$$y\left(x\right)=\int^{x}\frac{1}{\sqrt{h\left(\mu \right)}}d\mu .$$

We used the VST implemented in the R-package *DESeq2*, a model-based approach that relies on the variance-mean relation implied by a negative binomial distribution for the gene expression count data. The choice of transformation approach was motivated by properties of the clustering method, which often yields best results for (approximately) homoscedastic data, meaning that the variance of the variable, such as gene expression, does not depend on the mean. For RNA-seq count data, however, the variance typically increases with the mean. The commonly used procedure to handle this is to apply a logarithmic transform to the normalized count values after adding a small pseudo count. Unfortunately, now genes with low counts have a tendency to dominate the clustering result since they give the strongest signals in terms of relative difference between samples.

### Selection of Informative Genes

Our focus was to study how gene selection affects the clustering performance. For the considered data sets there are two “true” clusters defined by our gold standards. For supervised problems, where the class labels of the samples are known, feature selection is done by identifying a set of informative genes, in the simplest case, by applying a two-sample *t*-test to each gene and select the genes with the lowest *p*-values (Önskog et al., 2011). For cluster analysis problems, it can be argued that removing “non-informative” genes prior to the clustering will increase the clustering performance (Freyhult et al., 2010). Feature selection for cluster analysis is difficult for two reasons: (a) the sample labels are unknown and cannot be used to select informative genes, (b) in contrast to supervised classification it is not possible to use performance measures (e.g., error rates in classification) to compare and choose the best feature selection approach for the considered clustering problem.

We evaluated 13 different methods used for gene selection, where some are commonly used while others were included because they constitute principally different approaches. The methods ranked all genes based on how informative they were predicted to be, and the top ranked genes (100, 1000, or 3000 genes) were used in the downstream clustering. Hence, altogether 39 gene selection approaches were applied to the four data sets and evaluated against the gold standard.

The considered feature selection methods are motivated by fundamentally different ideas, which were used to group the methods into four categories. The four principles include selecting highly expressed genes, highly variably genes, highly correlated genes and genes with bi- or multimodal profiles. Below we give a general motivation behind the selection procedures within each group and a detailed description of the included methods.

One idea is to select genes with overall high expression values. Discriminating between disease subtypes can be difficult when the level of noise is high compared to the mean expression values, which makes it easier to detect differentially expressed genes among highly expressed genes. In this category of methods, we included the methods mean value (M) and third quartile (Q3).

Another group of methods is based on the spread of gene expression values across samples. Genes with large variability can contain interesting variations caused by disease subtype. In this category, we included the SD, the IQR, and the quadratic Rényi entropy (ENT).

A category involving correlation of genes includes a technique called co-expression (CoEx1) and a modified version (CoEx2). Tumor cells are under constant attack by the immune system and to survive, the genes must coordinate against the threat. Genes that are highly correlated to other genes may be involved in the same exposed networks and is therefore of interest as potential biomarkers. Co-expression among informative genes has been used for variable selection in clustering problems for high-dimensional microarray data (Wang et al., 2014).

**FIGURE 1:**
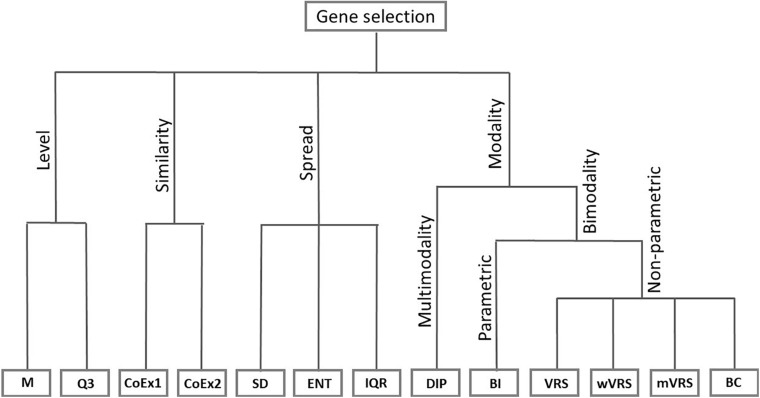
Feature selection methods divided into groups based on their properties. The included methods are mean value (M), third quartile (Q3), co-expression (CoEx1), modified co-expression (CoEx2), standard deviation (SD), interquartile range (IQR), entropy estimator (ENT), dip-test statistic (DIP), bimodality index (BI), variance reduction score (VRS), weighted variance reduction score (wVRS), modified variance reduction score (mVRS). and bimodality coefficient (BC).

Six of the methods studied in this article are based on the idea of modality. For informative genes, the distribution of gene expression among patients with different cancer subtypes can be expected to differ. It is therefore of interest to identify genes that have an expression distribution with more than one peak. We included the so-called dip-test (DIP), a method that identifies genes with multimodal distributions. In addition, we considered five methods that identify genes with bimodal distributions. We included the parametric method called the BI and four non-parametric methods: VRS, weighted variance reduction score (wVRS), modified variance reduction score (mVRS), and BC. The relationship between the considered gene selection methods is summarized in Figure 1.

#### The Mean Value Selection (M)

The mean value was calculated for each gene over all samples and the highest expressed genes were included in the analyses.

#### Third Quartile Selection (Q3)

Genes were arranged according to decreasing values of the third quartile and the genes with the highest Q3 values were selected.

#### The Standard Deviation Selection (SD)

The SD was calculated for each gene and the genes with the highest SDs were selected.

#### The Interquartile Range Selection (IQR)

The distance between the first and third quartile was calculated for each gene and genes with large distances were selected.

#### The Entropy Estimator Selection (ENT)

Entropy is an alternative to SD for measuring variability in gene expression across samples. Assuming the observed values *x*_1,_*x*_2,…,_*x*_*n*_ for a gene can be described by a distribution with density *f*(*x*), its quadratic Rényi entropy is defined as:

$$H_{2}\left(X\right)=-\log\left(\int f{\left(x\right)}^{2}dx\right).$$

To estimate this parameter, we use a non-parametric kernel-estimator (Gine and Nickl, 2008), obtained as

$$\text{ENT}=-\text{log}\left(\frac{2}{n\left(n-1\right)h}\sum_{i<j}K\left(\frac{x_{i}-x_{j}}{h}\right)\right).$$

The user specifies the kernel function *K*(⋅) and the bandwidth *h*. Here we employed the rectangular kernel, and for *h* we applied Silverman’s rule-of-thumb for kernel-density estimators and put $h=1.06\times \hat{\sigma }\times n^{-1/5}$, where $\hat{\sigma }$ is the sample SD. Genes with high entropy values were selected for the cluster analysis.

#### The Co-Expression Selection (CoEx1)

For each gene, the co-expressions to all other genes were calculated using Spearman correlation. Let *s*_*ij*_ = |ρ_*ij*_| denote the absolute value of the Spearman rank correlation ρ_*ij*_ between expression profiles for genes *i* and *j*. The matrix *S* with elements *s*_*ij*_ is considered as a similarity matrix for the genes with respect to co-expression. In the original article, the authors use Pearson correlation, but we applied Spearman correlation instead, which in earlier studies have proven to be more efficient in identifying co-expressed genes (Kumari et al., 2012; Wang et al., 2014). To rank genes according to their co-expression we define the *CoEx1* score for gene *i* as the median of the *s*_*ij*_ values, i.e.,

CoEx1i=medianjj≠i{sij}.

The genes with highest median correlations were selected.

#### The Modified Co-Expression Selection (CoEx2)

The co-expression network analysis was developed for variable selection in cluster analysis of microarray data. Since microarray data tend to be noisy, the authors argue that directly using the similarity matrix for co-expression analysis may be inappropriate and therefore suggests a transformation of the similarity matrix. The modified version uses a power transformation of the elements in the similarity matrix (Wang et al., 2014). The *CoEx2* score for gene *i* is defined as:

$$\text{CoEx}2_{i}=\sum_{j\neq i}s_{ij}^{3},$$

where *s*_*ij*_ are the elements of the similarity matrix. The genes with highest scores were selected for analysis.

#### The Dip-Test Statistic Selection (DIP)

The dip-test was used to test unimodality and is based on the maximum difference between the empirical distribution and the unimodal distribution that minimizes that maximum difference (Hartigan and Hartigan, 1985). Genes with low *p*-values were selected for analysis. The R-package *diptest* was used for calculations (Maechler, 2013).

#### The Bimodality Index Selection (BI)

For each gene, it is assumed that the density *f*(*x*) of the expression value can be described by a normal-mixture model with two components, i.e.,

$$f\left(x\right)=pN\left(\mu_{A},\sigma \right)+\left(1-p\right)N\left(\mu_{B},\sigma \right),$$

where μ_*A*_ and μ_*B*_ denote the mean in the two subgroups and *p* is the proportion of samples in one group (Wang et al., 2009). The BI is defined as

$$BI=\sqrt{p\left(1-p\right)}\frac{\left|\mu_{A}-\mu_{B}\right|}{\sigma }.$$

The expectation-maximization (EM) algorithm was used to estimate the BI using the R package *mixtools* (Benaglia et al., 2009). Ten different starting values were used for the EM-algorithm, generated from a grid with 10 values for the fraction parameter *p*, evenly spaced between 0 and 1, for more details, see Karlis and Xekalaki (2003). Genes with high BI were selected for analysis.

#### The Variance Reduction Score Selection (VRS)

The VRS is used for measuring the reduction of variance when splitting the data into two clusters (A and B) and is defined as the ratio of the within sum of squares (WSS) and the total sum of squares (TSS):

$$\text{VRS}=\frac{\text{WSS}}{\mathrm{TSS}}=\frac{\sum_{A}{\left(x_{i}-{\bar{x}}_{A}\right)}^{2}+\sum_{B}{\left(x_{i}-{\bar{x}}_{B}\right)}^{2}}{\sum_{i}{\left(x_{i}-\bar{x}\right)}^{2}},$$

where ${\bar{x}}_{A}$ and ${\bar{x}}_{B}$ denotes the mean values within group A and B. These values lie between zero and one, where a low score indicates an informative split (Hellwig et al., 2010). Hence, genes with a low score were selected for cluster analysis. The clusters were obtained using *k*-means clustering with *k* = 2.

#### The Weighted Variance Reduction Score Selection (wVRS)

The wVRS is a weighted version of VRS that takes sample size into account, i.e.,

$$\text{wVRS}=\frac{\frac{1}{2}\left(\frac{1}{n_{A}}\sum_{A}{\left(x_{i}-{\bar{x}}_{A}\right)}^{2}+\frac{1}{n_{B}}\sum_{B}{\left(x_{i}-{\bar{x}}_{B}\right)}^{2}\right)}{\frac{1}{n}\sum_{i}{\left(x_{i}-\bar{x}\right)}^{2}},$$

where *n_A_* and *n_B_* are the sample sizes in group A and B (Hellwig et al., 2010). The grouping of the data was obtained by the *k*-means algorithm, *k* = 2. Again, genes with a low score were selected.

#### The Modified Variance Reduction Score Selection (mVRS)

The mVRS considers the proportion of variance reduction when splitting data into two cluster by using the fuzzy *c*-means algorithm, also known as soft *k*-means clustering (Bezdek, 1981). Genes with a low score were selected for further analysis. The R-package *cluster* was used for calculations (Maechler et al., 2019).

#### The Bimodality Coefficient Selection (BC)

The BC yields a value between 0 and 1 (for large samples) and is calculated by

$$\text{BC}=\frac{\gamma^{2}+1}{\kappa +3\frac{{\left(n-1\right)}^{2}}{\left(n-2\right)\left(n-3\right)}},$$

where γ is the sample skewness, κ is the sample excess kurtosis and *n* is the sample size. The genes with largest BCs were selected for cluster analysis. The R-package *modes* was used for calculating the coefficient (Sathish and 4D Strategies, 2016).

### Evaluations

The considered gene selection approaches (13 methods times three levels of number of selected genes) were evaluated and compared to two negative controls (random selection and no selection) and a positive control (supervised selection).

#### Random Selection (RAND)

Here we randomly selected *k* genes, *k* = 100, 1000, or 3000. The performance of the random selection (RAND) was highly variable, therefore, the procedure was repeated 1000 times, resulting in 1000 performance measures. The evaluated gene selection methods were compared to the 25th, 50th, and 75th percentile and the mean value (RAND) of the random selection performance measures.

#### Supervised Selection (PVAL)

The gold standard partitions were used to rank genes according to how well they separated the two subtypes. A standard test for comparing two groups is the *t*-test, but for identification of differentially expressed genes it is common to use a generalized linear model (GLM). To describe the read count *K*_*ij*_ for gene *i* observed in sample *j*, we used a GLM from the negative-binomial (NB) family with a logarithmic link, given as:

$$K_{ij}∼\mathrm{NB}\left(\mathrm{mean}=\mu_{ij},\mathrm{dispersion}=\alpha_{i}\right),$$

$$\mu_{ij}=s_{ij}q_{ij},$$

$$\log_{2}q_{ij}=\beta_{i0}+\beta_{i1}x_{j}.$$

The normalizing factors *s*_*ij*_ compensate for differences in sequencing depth between samples and for eventual gene-related technical biases such as gene length. We used the default procedure where these factors are considered as fixed within each sample, *s*_*ij*_ = *s*_*j*_ and then only accounts for differences in sequencing depth between samples. These so-called size factors were estimated by the median-of-ratios method:

$$s_{j}={\text{median}}_{i:K_{i}^{R}\neq 0}\left(\frac{K_{ij}}{K_{i}^{R}}\right),K_{i}^{R}={\left(\prod_{j=1}^{n}K_{ij}\right)}^{1/n}$$

The linear part β_*i*0_ + β_*i*1_*x*_*j*1_ contains a categorical variable *x*_*j1*_ with two levels, corresponding to the cancer subgroups. The coefficient β_*i*1_ quantifies the extent to which gene *i* is differentially expressed between the groups. The intercept term β_*i*0_ models the base mean, which is allowed to differ between genes. The dispersion α_*i*_ was regarded as a gene-specific parameter in the model.

To fit the model (i.e., estimation of the parameters α_*i*_, β_*i*0_, β_*i*1_ for each gene *i*) we applied the R-package *DESeq2*, which implements the empirical Bayes shrinkage method (Love et al., 2014). The *p*-value for the test that gene *i* is differently expressed (i.e., *H*_0_ : β_*i*1_ = 0) was then used to rank genes, so that genes with lowest *p*-values were used for clustering. The method was applied to data that had been filtered for low expressed genes, but not normalized using the variance stabilizing transform.

#### No Selection (ALL)

Gene selection is performed to remove non-informative and irrelevant genes. An alternative is to base the clustering on all genes, and we included the case of no selection as a reference point.

#### Similarity Between Feature Selection Methods

Each selection procedure was characterized by calculating the mean value and SD of the selected genes. Procedures with similar characteristics may also make similar selections. In addition, we carried out a more direct analysis by measuring the overlap between the approaches, i.e., for each pair of approaches we measured the percentage of genes selected by both methods.

#### Performance of the Feature Selection Methods

The clustering performance was measured using the ARI based on the clustering result compared to the gold standard partition. An ARI-value of 1 indicates a complete match to the gold standard partition, whereas a value of 0 indicates an agreement as good as a random clustering.

#### Detailed Evaluation of Top-Performing Feature Selection Methods

The evaluations described above utilize four data sets and were used to identify a set of interesting feature selection methods. To deeper understand our findings, we used these data sets to simulate two types of data sets using stratified subsampling with replacement from the original data: balanced data sets where 50 samples were drawn from each subtype and skewed data sets were 25 (75) of the samples were drawn from the least (most) common subtype. Hundred data sets were sampled for each type. The chosen selection methods were applied to each of the simulated data sets, hierarchical clustering with Euclidean distance, was performed on the top 1000 ranked genes to cluster the samples in two groups and ARI was used to measure the performance. For each pair of methods, the pairwise ARI-observations were used to construct differences and the one sample *t*-test was used to test if the expected value of the difference deviated from zero.

In addition to the ARI-values we also observed the number of samples in the smallest of the two groups generated by the clustering, i.e., a number between 1 and 50. Again, the one sample *t*-test was used to investigate differences between the considered selection methods. Since BI is computationally heavy, the EM-algorithm was used with only one initial value of the parameter vector, obtained as follows: first the data was divided into two groups using the *k*-means (*k* = 2) clustering algorithm, and then the means, SDs and size fraction in the two subsamples were used as starting values for the mixing parameters.

For the top-performing feature selection methods, we also investigated the change in ARI-values when increasing the number of selected genes in the cluster analysis. The number of selected genes was increased gradually in 1000 steps between two selected genes up to all genes remaining after initial filtration. Since the clustering result is highly variable, we applied a running mean over 100 values to get a smoother curve.

The aim of the feature selection is to exclude genes that are non-informative for distinguishing between the disease subtypes. As a way of measuring the relevance of the selected features, the list of 1000 top scoring genes were compared to 299 known cancer driver genes (Bailey et al., 2018). Enrichment of genes relevant to cancer etiology were tested using a one sided Fisher’s exact test.

## Results

We tested the performance of 13 feature selection methods when identifying subgroups using cluster analysis on four human cancer data sets. For each method the *k* top ranked genes were selected, *k* = 100, 1000, and 3000. Three references were considered: a negative control where all genes were selected, a negative control where *k* genes were randomly selected and a positive control where genes were selected using a supervised approach. The selection methods were applied on the 15,298, 15,388, 15,397, and 15,397 genes that remained after filtering low expressed genes in KIRP, STAD, LGG, and BRCA, respectively. In addition, a small simulation study was performed with the objective to investigate how clustering is affected when non-informative features are included in the analysis.

### Simulation Study

In the case when the clustering was based on only the informative features all the clustering results were identical to the desired AB-partition with an average ARI equal to 1. In the case when all features were included, the average ARI was 0.34. For the case when the clustering was based on the features with the highest SDs the clustering performance peaked when around 600 features were included and declined when more features were added, see Supplementary Figure 1. Although the negative effect of including non-informative features is likely to be general, it should be stressed that the magnitude of the effect depends on the effect size, the sample size, and the percentage of informative features. Moreover, in real problems we may in addition to informative and non-informative features have features that are informative to secondary factors, e.g., gender, age, and prior treatments.

### Characteristics of Top Ranked Genes

As an initial investigation, we studied the mean expression and SD of top ranked genes obtained for the considered feature selection approaches.

#### The Mean Value of Selected Genes

**FIGURE 2:**
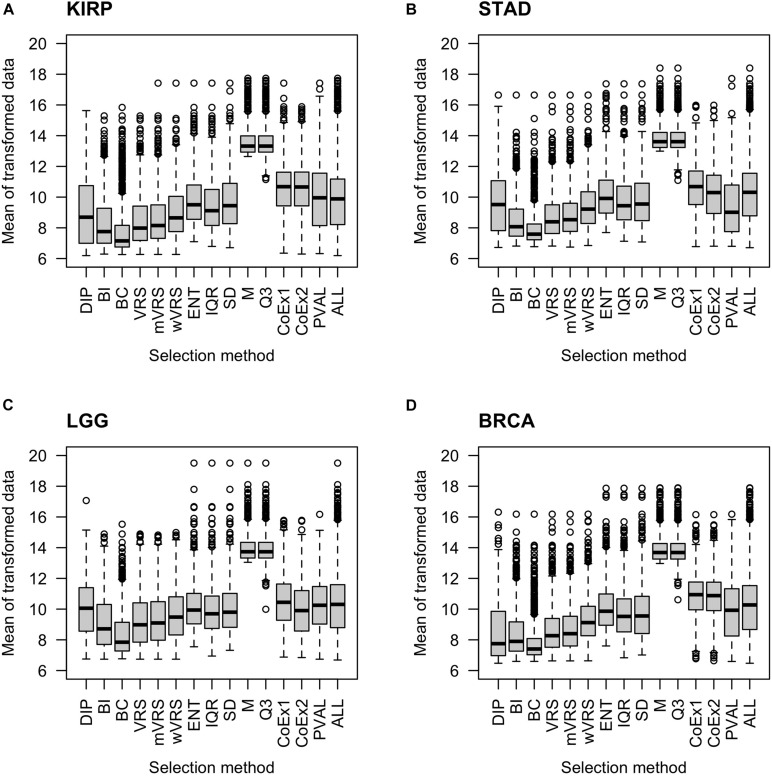
Boxplots of mean expression values over all samples for 1000 selected genes. The figure shows the result for the data sets KIRP **(A)**, STAD **(B)**, LGG **(C)**, and BRCA **(D)**. Each plot displays expression values of preprocessed data for the 13 feature selection methods, the positive control (PVAL) and the negative control (ALL) including all genes. The gene selection methods are: dip-test statistic (DIP), bimodality index (BI), bimodality coefficient (BC), variance reduction score (VRS), modified variance reduction score (mVRS), weighted variance reduction score (wVRS), entropy estimator (ENT), interquartile range (IQR), standard deviation (SD), mean value (M), third quartile (Q3), co-expression (CoEx1), and modified co-expression (CoEx2).

As expected, genes selected using the median (M) or the third quartile (Q3) were highly expressed compared to the other methods. The BC approach selected genes expressed at a very low level. The supervised approach (PVAL) selected genes at an intermediate gene expression level, which was comparable to the expression level seen in the whole data (ALL). Approaches using SD, IQR, ENT, CoEx1, and CoEx2 selected genes with mean gene expression similar to that obtained by the supervised approach. The remaining methods, BI, the dip-test statistic (DIP) and the variance reduction scores (VRS, wVRS, and mVRS), selected genes expressed at a relatively low level. Interestingly, the same relative patterns were observed for all data sets and independently of the number of selected genes, see Figure 2 and Supplementary Figures 2, 3.

#### The Standard Deviation of Selected Genes

**FIGURE 3:**
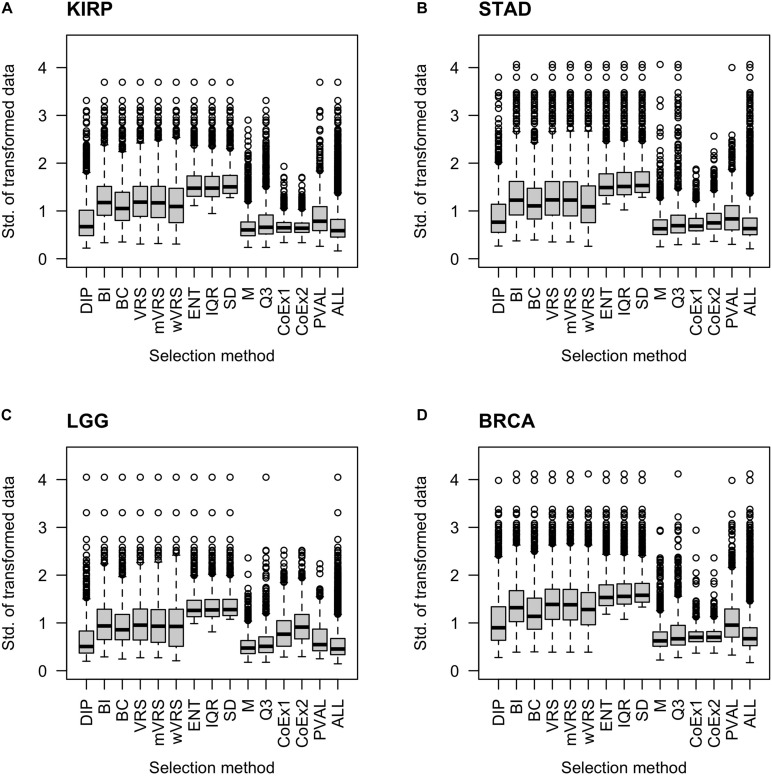
Boxplots of standard deviation across samples for 1000 selected genes. Each plot displays standard deviation based on preprocessed data for the 13 feature selection methods, the positive control (PVAL) and the negative control (ALL) including all genes. The figure shows the result for the data sets KIRP **(A)**, STAD **(B)**, LGG **(C)**, and BRCA **(D)**. The gene selection methods are: dip-test statistic (DIP), bimodality index (BI), bimodality coefficient (BC), variance reduction score (VRS), modified variance reduction score (mVRS), weighted variance reduction score (wVRS), entropy estimator (ENT), interquartile range (IQR), standard deviation (SD), mean value (M), third quartile (Q3), co-expression (CoEx1), and modified co-expression (CoEx2).

It is natural to assume that informative genes should have relatively high SD, compared to most other genes. As expected, genes selected using SD, ENT, and IQR, had high SDs. The M and Q3 methods selected genes with relatively low variation, which was close to the SD observed in the whole data sets (ALL). Intermediate values of SD were observed among genes selected using the variance reduction scores (VRS, wVRS, and mVRS), and BI. For BC, DIP, CoEx1, CoEx2, and the supervised approach (PVAL), the level of SD varied between low and intermediate depending on data set and number of selected genes, see Figure 3 and Supplementary Figures 4, 5.

#### Overlap of Selected Genes

**FIGURE 4:**
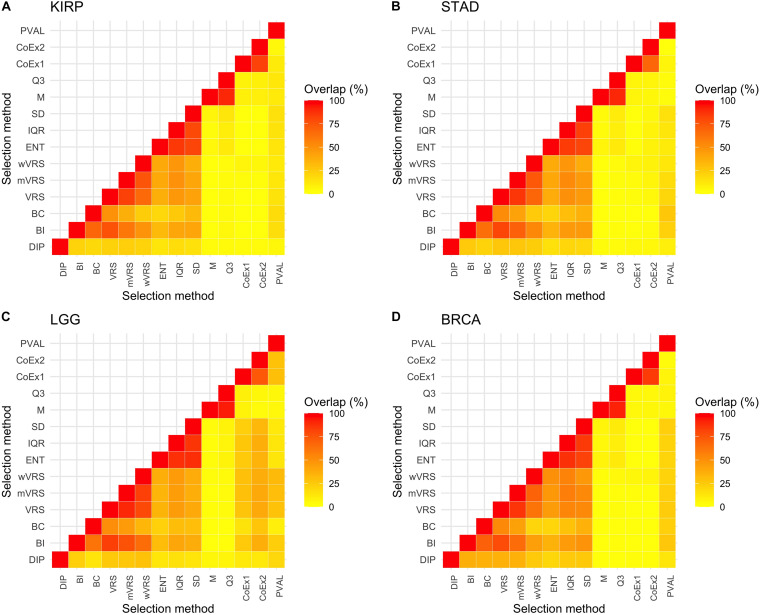
Percentage overlap between 1000 selected genes for the 13 different feature selection methods and the four data sets KIRP **(A)**, STAD **(B)**, LGG **(C)**, and BRCA **(D)**. The feature selection methods are: dip-test statistic (DIP), bimodality index (BI), bimodality coefficient (BC), variance reduction score (VRS), modified variance reduction score (mVRS), weighted variance reduction score (wVRS), entropy estimator (ENT), interquartile range (IQR), standard deviation (SD), mean value (M), third quartile (Q3), co-expression (CoEx1), modified co-expression (CoEx2), and the positive control (PVAL).

The above results show that methods based on similar selection principles also have similar properties with respect to the mean and SD of the selected genes, see Figures 2, 3. Next, we investigated to what degree the methods selected the same genes, by studying the overlap when 1000 genes were selected. The overlap between M and Q3 was high (>90%) in all data sets, but both methods showed very limited resemblance to the other methods (<6% overlap in average). High agreement was also observed between SD, ENT and IQR (85% in average), as well as between CoEx1 and CoEx2 (77% in average). CoEx1 and CoEx2 showed low overlap with the remaining methods (<10%). The intersection between VRS, mVRS and wVRS was in average 73% in all data sets, and the group showed a greater resemblance to BI than to BC (in average 67 vs 40%), see Figure 4. The positive control (PVAL), that is expected to be a good selection procedure, had a very small overlap with the methods M, Q3, CoEx1, and CoEx2. For detailed results, see Figure 4.

### Feature Selection Methods

**FIGURE 5:**
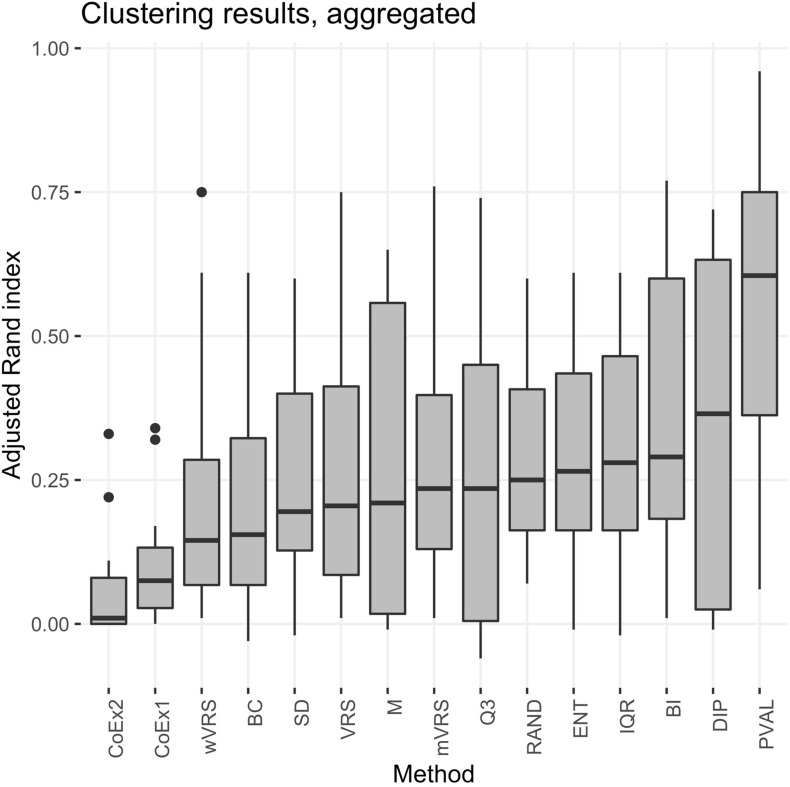
Boxplot of aggregated clustering performance over the four data sets KIRP, STAD, LGG, and BRCA. Performance is measured using adjusted Rand index and the feature selection methods are ordered according to increasing median values. The selection methods are: dip-test statistic (DIP), bimodality index (BI), bimodality coefficient (BC), variance reduction score (VRS), modified variance reduction score (mVRS), weighted variance reduction score (wVRS), entropy estimator (ENT), interquartile range (IQR), standard deviation (SD), mean value (M), third quartile (Q3), co-expression (CoEx1), modified co-expression (CoEx2), the positive control (PVAL), and the negative control (RAND).

The performance was measured using the adjusted Rand index (ARI) comparing the obtained clustering result with the gold standard. Each of the 13 selection methods were used to cluster the four cancer data sets by selecting the 100, 1000, or 3000 top ranked genes. Hence, each method was used to perform 12 cluster analyses and generated 12 ARI-values. The results were compared to two negative controls (randomly selected genes and a selection including all genes) and a positive control (PVAL). As expected, the supervised selection approach (PVAL) had the highest combined performance (considering the median value of the 12 ARI-values) followed in decreasing order by DIP, BI, IQR, ENT, RAND, Q3, mVRS, M, VRS, SD, BC, wVRS, CoEx1, and CoEx2, see Figure 5. However, the relative performance of the methods varied between the four data sets and was also affected by the number of selected genes. Evaluating the approaches based on their mean ranking taken over all 12 analyses revealed that the supervised approach performed best followed by BI, mVRS, DIP, VRS, RAND, IQR, ENT, Q3, M, SD, wVRS, BC, CoEx1, and CoEx2, see Table 1.

**TABLE 1:** Rank of feature selection methods for data sets KIRP, STAD, LGG, and BRCA based on adjusted Rand index.

	DIP	BI	BC	VRS	mVRS	wVRS	ENT	IQR	SD	M	Q3	CoEx1	CoEx2	PVAL	RAND
KIRP100	14	2	3.5	6	3.5	12	7.5	9	10	7.5	5	13	15	1	11
STAD100	7	4.5	8	4.5	6	1	13	15	14	10	12	11	9	3	2
LGG100	14	10	10	10	10	10	7	5	6	15	13	4	3	1	2
BRCA100	6	2	8.5	5	3	4	7	8.5	10	11	13	14	15	1	12
KIRP1000	6.5	1	10.5	8	6.5	13	4.5	2.5	9	4.5	2.5	14	15	10.5	12
STAD1000	2	3	14	11	6	9	7	4	5	12	15	10	13	1	8
LGG1000	2	10	13	14	9	15	11	7	8	3	4	6	12	1	5
BRCA1000	5	8.5	6.5	1.5	3	6.5	11.5	11.5	13	10	4	14	15	1.5	8.5
KIRP3000	7	1	12	6	11	9.5	3.5	3.5	3.5	13	9.5	14	15	3.5	8
STAD3000	13.5	1	6	5	8	9	4	3	12	13.5	15	10	11	2	7
LGG3000	2	9	15	13	8	14	11.5	10	11.5	6	3	7	5	1	4
BRCA3000	6	11.5	11.5	3.5	3.5	11.5	6	9	6	2	1	14	15	11.5	8
Mean rank	7.1	5.3	9.9	7.3	6.5	9.5	7.8	7.3	9.0	9.0	8.1	10.9	11.9	3.2	7.3

*The table shows results for selection of top ranked genes at three levels: 100, 1000, and 3000 genes. The gene selection methods are: dip-test statistic (DIP), bimodality index (BI), bimodality coefficient (BC), variance reduction score (VRS), modified variance reduction score (mVRS), weighted variance reduction score (wVRS), entropy estimator (ENT), interquartile range (IQR), standard deviation (SD), mean value (M), third quartile (Q3), co-expression (CoEx1), modified co-expression (CoEx2), and the positive control (PVAL).*

**FIGURE 6:**
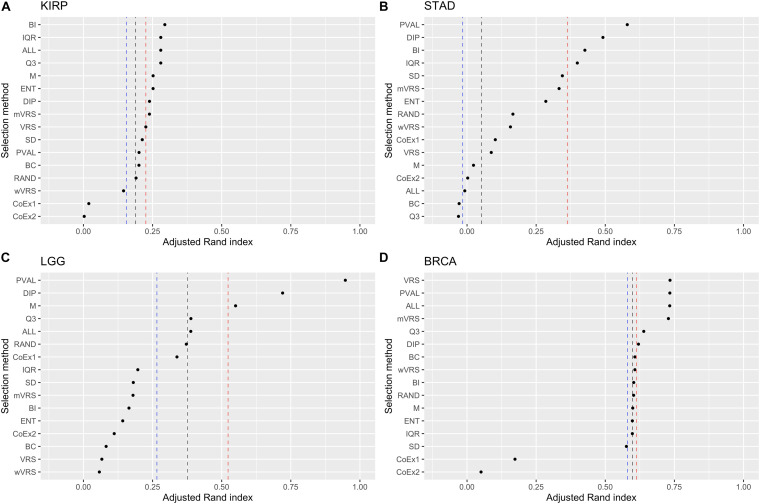
Adjusted Rand index for hierarchical clustering using the top 1000 ranked genes on the datasets KIRP **(A)**, STAD **(B)**, LGG **(C)**, and BRCA **(D)**. The vertical lines represent the first, second, and third quartiles of a random selection. The feature selection methods on the *y*-axis are: dip-test statistic (DIP), bimodality index (BI), bimodality coefficient (BC), variance reduction score (VRS), modified variance reduction score (mVRS), weighted variance reduction score (wVRS), entropy estimator (ENT), interquartile range (IQR), standard deviation (SD), mean value (M), third quartile (Q3), co-expression (CoEx1), modified co-expression (CoEx2), the positive control (PVAL), and the two negative controls ALL and RAND. The selection methods are ordered according to increasing performance.

Ranking genes according to Q3 or M is a simple way of selecting highly expressed genes. The performance for Q3 and M varied from being top performing (BRCA 3000 genes) to be at the very bottom (STAD 3000 genes). In KIRP, Q3 was always ranked higher than M and for STAD it was the other way around. For LGG and BRCA it varied depending on number of features included, see Figure 6 and Supplementary Figures 6, 7. Altogether, Q3 performed slightly better than M.

Of the three methods relying on variability across samples, IQR and ENT generally performed better than the commonly used SD procedure. IQR outperformed ENT in the LGG data, while ENT performed better on the BRCA data. In KIRP and STAD it depended on the number of included features, see Figure 6 and Supplementary Figures 6, 7. Within this category, IQR performed best and should be considered as a simple alternative to SD.

The methods relying on gene correlation (CoEx1 and CoEx2), performed worst of all considered methods, with CoEx2 slightly worse than CoEx1, see Table 1.

Among methods based on modality (i.e., DIP, BI, VRS, mWRS, wVRS, and BC), BI and DIP where the methods with the overall highest performance. The relative performance of DIP was particular good when more genes were selected (1000 or 3000), while BI performed particularly well on the KIRP and STAD data, see Table 1, Figure 6, and Supplementary Figures 6, 7. When 1000 genes were selected, the overlap between DIP and BI varied between 21 and 37%, see Figure 4. Furthermore, DIP tended to select genes that were slightly higher expressed than BI, see Figure 2 and Supplementary Figures 2, 3. More evident, BI selected genes with higher SD than DIP, see Figure 3 and Supplementary Figures 4, 5. Altogether, this suggests that although performing similar, and relatively well, DIP and BI select rather different genes with different characteristics.

### Comparisons to Positive and Negative Controls

Intuitively, selecting the *k* top scoring genes using a good feature selection method should in average result in a better clustering performance than obtained when randomly selecting *k* genes, but worse performance than using a supervised approach. However, if the gene expressions are highly influenced by a *secondary factor* (i.e., a factor that is not informative for predicting the subgroups) applying feature selection may result in a performance worse than the random selection.

As expected, the supervised approach PVAL was commonly superior to the unsupervised selection approaches, although occasionally performed slightly worse than some other methods, see Table 1. For the LGG data randomly selecting *k* genes outperformed most of the selection methods, see Figure 5 and Supplementary Figures 6, 7. This may indicate that the RNA-expression of the individuals is influenced by secondary factors or that the binary partitions defined by the gold standard are heterogeneous and preferably should be divided further.

An alternative to applying feature selection is to include all genes in the cluster analysis for which the ARI-values 0.28, −0.01, 0.39, and 0.73 were observed for KIRP, STAD, LGG, and BRCA, respectively. For KIRP and BRCA, including all genes was as good as the best performing selection methods, but for STAD and LGG the best selection methods yielded considerably higher ARI-values, 0.66 and 0.72, respectively, see Table 2. On the other hand, variable selection often resulted in lower ARI-values compared to including all genes, in particular when just 100 genes were selected, see Table 2. This suggests that variable selection has potential to improve the clustering, but that the choice of methods and the number of selected genes are crucial for the performance.

**TABLE 2:** Adjusted Rand index for 13 feature selection methods, a negative (RAND) and positive control (PVAL) for data sets KIRP, STAD, LGG, and BRCA.

	DIP	BI	BC	VRS	mVRS	wVRS	ENT	IQR	SD	M	Q3	CoEx1	CoEx2	PVAL	RAND
KIRP100	0.01	0.25	0.23	0.18	0.23	0.10	0.17	0.14	0.14	0.17	0.19	0.05	0.00	0.39	0.11
STAD100	0.03	0.05	0.03	0.05	0.04	0.07	−0.01	−0.02	−0.02	0.01	−0.01	0.01	0.01	0.06	0.07
LGG100	0.00	0.01	0.01	0.01	0.01	0.01	0.08	0.10	0.09	0.00	0.01	0.12	0.22	0.96	0.29
BRCA100	0.67	0.77	0.61	0.75	0.76	0.75	0.61	0.61	0.60	0.58	0.37	0.11	0.07	0.78	0.49
KIRP1000	0.24	0.29	0.20	0.23	0.24	0.15	0.25	0.28	0.21	0.25	0.28	0.02	0.00	0.20	0.19
STAD1000	0.49	0.43	−0.03	0.09	0.33	0.16	0.28	0.40	0.34	0.02	−0.03	0.10	0.00	0.58	0.17
LGG1000	0.72	0.16	0.08	0.07	0.18	0.06	0.14	0.20	0.18	0.55	0.39	0.34	0.11	0.95	0.37
BRCA1000	0.62	0.60	0.61	0.73	0.73	0.61	0.60	0.60	0.58	0.60	0.64	0.17	0.05	0.73	0.60
KIRP3000	0.21	0.29	0.12	0.27	0.16	0.18	0.28	0.28	0.28	0.11	0.18	0.03	0.00	0.28	0.21
STAD3000	−0.01	0.66	0.19	0.35	0.04	0.02	0.38	0.42	−0.01	−0.01	−0.06	0.00	0.00	0.61	0.14
LGG3000	0.71	0.19	0.08	0.15	0.27	0.14	0.17	0.17	0.17	0.32	0.63	0.32	0.33	0.74	0.38
BRCA3000	0.60	0.60	0.60	0.60	0.60	0.60	0.60	0.60	0.60	0.65	0.74	0.04	0.01	0.60	0.60

*The table shows results for selection of top ranked genes at three levels: 100, 1000, and 3000 genes. Adjusted Rand index when including all genes was obtained as 0.28, −0.01, 0.39, and 0.73 for KIRP, STAD, LGG, and BRCA, respectively. The gene selection methods are dip-test statistic (DIP), bimodality index (BI), bimodality coefficient (BC), variance reduction score (VRS), modified variance reduction score (mVRS), weighted variance reduction score (wVRS), entropy estimator (ENT), interquartile range (IQR), standard deviation (SD), mean value (M), third quartile (Q3), co-expression (CoEx1), and modified co-expression (CoEx2).*

### Detailed Evaluation of Top-Performing Feature Selection Methods

Based on our findings we conclude that DIP, BI, and mVRS are the most promising methods and that good performance is usually obtained when 1000 genes are selected. These methods also ranked high when *k*-means (*k* = 2) clustering and hierarchical clustering with a correlation-based distance measure were used, see Supplementary Tables 1–4. DIP, BI, and mVRS together with the commonly used SD method were therefore selected for a deeper study based on hundreds of simulated balanced and skewed data sets, see section “Detailed Evaluation of Top-Performing Feature Selection Methods.”

**FIGURE 7:**
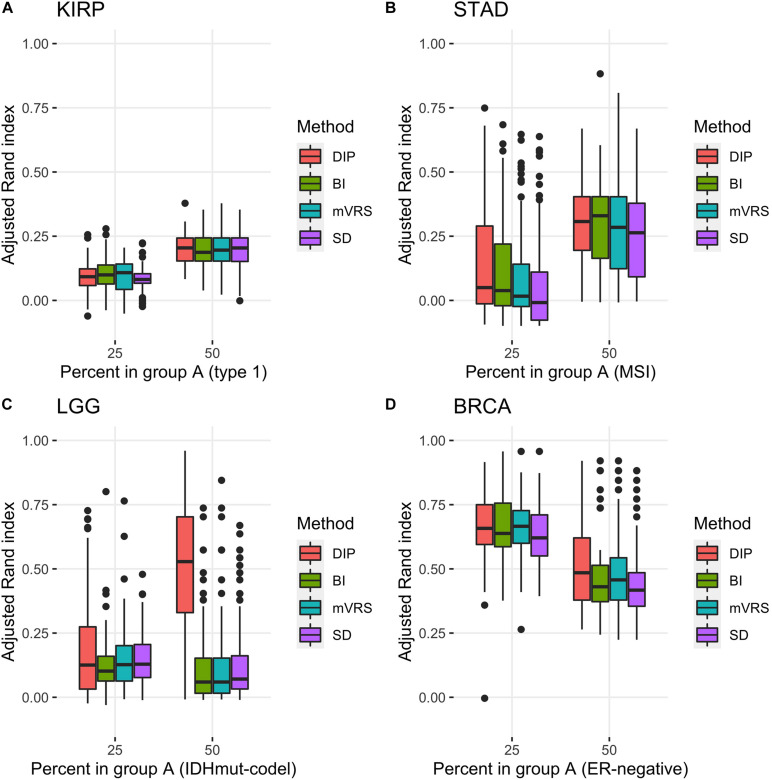
Adjusted Rand index for clustering based on 1000 selected genes with different proportions of the subtypes based on 100 random samplings of the KIRP **(A)**, STAD **(B)**, LGG **(C)**, and BRCA **(D)** data. The figure shows results for unbalanced data with 25 (75) samples in the smaller (larger) subgroup and for balanced data sets with 50 samples in each subgroup. The selection methods on the *x*-axis are the dip-test statistic (DIP), bimodality index (BI), the modified variance reduction score (mVRS), and standard deviation (SD).

Pairwise comparisons with respect to ARI between DIP, BI, mVRS, and SD showed that DIP was as good or better than the other methods, with the exception that BI was slightly better than DIP for the skewed KIRP data set, see Table 3 and Figure 7. Furthermore, SD did not perform well and was the worst performing method for most of the simulations, Table 3. For LGG, STAD, and BRCA the difference in average ARI between DIP and SD ranged between 0.03 and 0.35 and five out of six findings were significant, see Table 3.

**TABLE 3:** The mean value of 100 pairwise adjusted Rand index-differences (row method – column method) for different pairs of feature selection methods: the dip-test (DIP), bimodality index (BI), modified variance reduction score (mVRS) and standard deviation (SD).

		25%			50%		
		BI	mVRS	SD	BI	mVRS	SD
KIRP	DIP	–0.01	0.00	0.01	0.00	0.00	0.01
	BI		0.01	0.02*		0.00	0.01
	mVRS			0.01			0.01
STAD	DIP	0.05	0.07**	0.10***	0.00	0.02	0.05**
	BI		0.02	0.06**		0.02	0.06**
	mVRS			0.03			0.03
LGG	DIP	0.06**	0.04	0.04	0.37***	0.37***	0.35***
	BI		−0.03*	−0.02*		0.00	–0.02
	mVRS			0.00			–0.02
BRCA	DIP	0.01	0.00	0.03**	0.06***	0.02	0.07***
	BI		–0.01	0.03***		−0.03*	0.01
	mVRS			0.03**			0.05***

Simulations were made for the data sets KIRP, STAD, LGG, and BRCA, and two types of data sets were simulated: unbalanced data where 25% of the individuals belonged to the minor class and a balanced data set were 50% of the individuals belonged to each of the two classes. The number of samples was 100 in each simulation. The one sample t-test was used to test if the mean difference deviated from zero. Positive (negative) differences indicate that the row-method was better (worse) than the column method. Here *, **, ** denote a significant result at the 0.05, 0.01, and 0.001 significance level, respectively.

In order to better understand the results, we investigated the *number of samples in the smallest group* (NSSG) obtained doing a cluster analysis resulting in two groups. This number should be close to 50 for the balanced data set and close to 25 for the skewed data sets. Moreover, if this number is very small it indicates that the clustering is governed by just a few samples (outliers). Interestingly, the ARI-differences and NSSG-differences were correlated, so that methods with relatively high ARI also had a relatively high NSSG, see Table 3 and Supplementary Table 5. In particular SD had considerably lower NSSG than the other methods, especially for the balanced data, see Supplementary Table 5.

**FIGURE 8:**
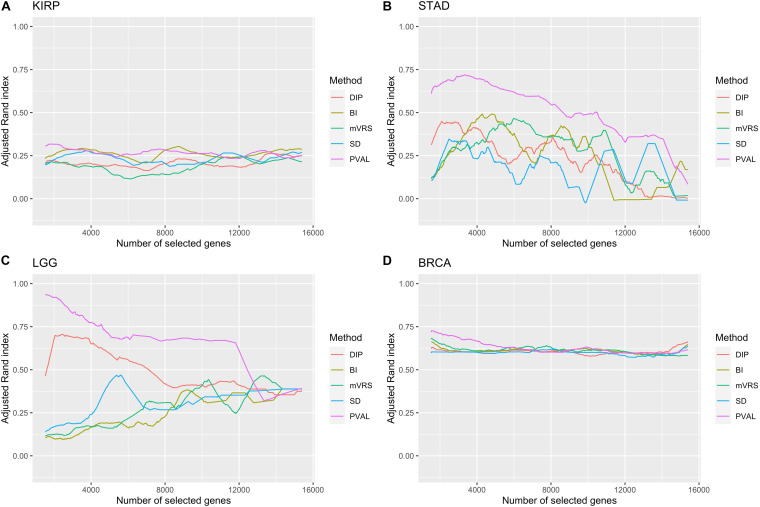
Adjusted Rand index for a gradual increase in number of selected genes. The number of included genes was increased over a 1000 steps from 2 to up to all genes remaining after filtration. Of low expressed genes. The feature selection methods are: dip-test statistic (DIP), bimodality index (BI), modified variance reduction score (mVRS), standard deviation (SD), and the positive control (PVAL). The performance is shown for the KIRP **(A)**, STAD **(B)**, LGG **(C)**, and BRCA **(D)** data. A running mean over 100 values was applied to get a smoother curve.

It is not trivial to select how many genes to include in the cluster analysis. The results from the analysis of ARI in relation to the number of selected genes showed a highly variable performance, especially in STAD and LGG, see Figure 8. The most noticeable result was the gradual decrease in performance in the LGG data for DIP and PVAL when including more genes, indicating that it is possible to increase the ability to identify disease subgroups substantially when choosing features wisely. For BI, mVRS, and SD in the LGG data, the general trend was an increase in performance when including more genes. In STAD, the general trend was a decreasing performance when including more genes. In both KIRP and BRCA the performance was relatively stable when changing the number of included genes. At least for BRCA, this might be explained by the high number of informative genes.

The overlap between the 299 cancer driver genes and all genes remaining after initial filtration were 279, 285, 279, and 286 for KIRP, STAD, LGG, and BRCA, respectively. No enrichment of cancer driver genes was observed for the 1000 top ranked genes for DIP, BI, mVRS, and SD, except for in BRCA where genes selected using SD had a significantly higher proportion of cancer driver genes (*p* < 0.001), see Table 4. When extending the comparison to include all 13 selection methods, a significant overrepresentation of cancer driver genes was observed for both M and Q3 (data not shown) in all four data sets, suggesting that the detected cancer driver genes are generally high expressed.

**TABLE 4:** Proportion of cancer driver genes among the 1000 top ranked genes.

	DIP	BI	mVRS	SD	PVAL
KIRP	0.013 (0.92)	0.011 (0.98)	0.011 (0.98)	0.013 (0.92)	0.009 (0.99)
STAD	0.018 (0.59)	0.015 (0.84)	0.015 (0.84)	0.023 (0.17)	0.021 (0.31)
LGG	0.021 (0.27)	0.016 (0.73)	0.015 (0.81)	0.013 (0.92)	0.021 (0.27)
BRCA	0.017 (0.68)	0.018 (0.59)	0.016 (0.77)	0.032 (0)	0.024 (0.12)

*The proportions in the entire data setswere obtained as 0.018, 0.019, 0.018, and 0.019 for KIRP, STAD, LGG, and BRCA, respectively. The p-value from a one sided Fisher’s exact test is given within paranthesis.*

## Discussion

Feature selection prior to clustering RNA-seq is common and is often done by selecting the genes with the highest SD (i.e., the SD method). However, this problem has not been well studied and there is little evidence that selecting genes with high SD is the best approach. Before we discuss our findings, it is worth pointing out that measuring the performance of feature selection methods is difficult. The clustering performance, in our case measured using ARI-values, does in addition to the feature selection algorithm also depend on clustering method, the nature of the data and how the gold standard is defined. Samples from a cancer cohort can be divided in several logical partitions, e.g., partitions defined by gender, age or disease subtype. For these partitions, it is likely that a set of genes will be differentially expressed between the groups. Hence, a low ARI-value does not automatically mean that the cluster analysis failed, it can also be a consequence of secondary factors affecting the data or that the groups defined by the gold standards are heterogeneous and should be further divided.

The general idea behind feature selection prior to cluster analysis is to remove genes that do not contain information about the “true partition” of the samples, e.g., genes that are identically distributed among all samples and therefore only contribute with noise, making the analysis harder.

In the considered simulation study only a small set of the genes were informative and including all genes in the analysis had a negative effect on the clustering result. This negative effect will be reduced when the number of informative genes increases (data not shown). RNA-seq cancer data are much more complex than the simulated data and the informative genes are unknown although they in our case can be predicted using a supervised test. For the LGG and STAD data considerably better clustering results were obtained when genes predicted to be informative were used compared to when all genes were included, which suggests that feature selection has the potential to improve the clustering performance. For BRCA and KIRP, the gain of using supervised selection was limited, which suggests that feature selection is unlikely to have a positive effect on the clustering result. Arguably, a feature selection approach should identify informative genes related to the factor of interest.

For the considered four data sets, there were feature selection approaches that either were equally good or considerably better than including all genes, which again suggests that feature selection has potential. For example, for the STAD data, including all genes resulted in a partition no better than expected by *chance* (i.e., ARI close to 0) while the best feature selection approach resulted in a partition highly correlated with the partition defined by the gold standard (ARI = 0.66). On the other hand, applying feature selection often resulted in a lower performance than including all genes in the analysis, which suggests that the choice of feature selection method and the number of selected genes are important.

We included 13 variable selection methods that theoretically and methodologically can be grouped in four fundamentally different groups: methods that select highly expressed genes (M and Q3), methods that select highly variable genes (ENT, IQR, and SD), methods that select highly correlated genes (CoEx1 and CoEx2) and methods that select genes with respect to modality (BI, BC, DIP, mVRS, VRS, and wVRS). The correlation-based methods had surprisingly low performance, often worse than by selecting genes randomly. These methods were developed for variable selection in microarray data, which might explain the poor performance and suggest that these methods need to be modified for RNA-sequence data. Since CoEx1 and CoEx2 select genes with a relatively low SD across samples (Figure 3), a hybrid method that combines correlation and a bimodality score or measure of spread could be worth to investigate further. Selecting highly expressed genes is motivated by the fact that the signal to noise ratio is believed to be relatively high for highly expressed genes. Hence, by including highly expressed genes we get less noisy data and thereby better results. These methods, in particular Q3, worked surprisingly well and commonly better than selecting genes randomly. Although these methods did not perform as well as the best selection methods, the results suggest that they may work well in combined approaches as discussed at the end of this section.

An important finding is that the commonly used SD method did not perform well. One reason for this may be that SD compared to other methods is more likely to include genes with outliers and extreme values. Samples with extreme values can govern the clustering and incorrectly result in a binary clustering where the smaller of the two groups contains a low number of individuals. The results showed that SD indeed had fewer samples in the minority group compared to DIP, BI, and mVRS, which may explain the ARI-results. Furthermore, the IQR that is a robust alternative to SD performed better than SD.

The best performing selection methods BI, DIP, and mVRS all aim to identify genes based on modality. With the exception of DIP, these methods strive to detect genes with a clear bi-modality pattern, while DIP is more general a search for multimodality patterns. For the case where 1000 genes were selected, DIP achieved the best performance and worked well for both balanced and skewed data sets. Interestingly, DIP had compared to BI and mVRS often more samples in the minority group obtained from the binary clustering.

For the LGG data with 1000/3000 selected genes, both the original data and the simulated data sets, DIP performed considerably better than BI and mVRS, which in turn performed worse than a strategy including all genes. Furthermore, the overlap between the genes selected by DIP and the two other methods was small, much smaller than observed for the other data sets, see Figure 4. This may indicate that the partition defined by the gold standard should be further divided, which in turn explain why methods searching for genes with a bimodal pattern fails. In general, all methods aiming to identify bimodality will suffer if the partitioning of interest consists of more than two groups, in particular if there exist one or more secondary factors that define two groups, e.g., gender.

Some potential secondary factors and their partitions are sometimes known, e.g., the age and gender of the patients, prior treatments and technical design questions, e.g., which hospital analyzed the samples. This information can in principle be used when selecting the genes, e.g., by omitting genes that are highly correlated to any of the known secondary factors. Another way to improve feature selection may be to combine two or more approaches, e.g., demand that the selected genes are both highly expressed and have high DIP scores. How to include additional meta information and combine different selection methods are open questions that requires more research.

For most of the feature selection methods, the overlap between selected genes and previously identified cancer driver genes was relatively low. Lists of candidate cancer driver genes are continually updated as new discoveries are made and there are several published lists of genes that are important for cancer development. Comparing against alternative gene lists may affect the results. Moreover, many of the considered cancer driver genes are affected by cancer in general but are not necessarily informative for the partition of interest. We did observe an enrichment of cancer driver genes among the set of genes selected using M and Q3, suggesting that the confirmed cancer driver genes are in general expressed at higher levels.

Here *k*-means (*k* = 2) and hierarchical clustering with Ward’s linkage and either the Euclidean distance or a correlation-based distance were used to cluster the samples. It should be stressed that the choice of clustering method may affect the relative performance of the considered feature selection methods. The choice was motivated by prior findings and since these approaches are widely used. The number of selected genes were 100, 1000, or 3000 and these choices were based on our prior experience (Vidman et al., 2019 and Freyhult et al., 2010). However, how to determine the optimal number of genes to include is an open question that needs more research. These choices affect the ARI-values and may also have an effect on the relative performance of the considered feature selection methods. Moreover, the performance of any clustering approach, including pre-processing, standardization, feature selection, and the clustering, is highly dependent on the data making it difficult to give general advices. Nevertheless, the results presented in this article suggest that variable selection using DIP with 1000 selected genes is a good choice and considerably better than selecting genes based on the observed SD.

The study focuses on the relative merits of feature selection strategies commonly categorized as filtering methods in the literature, and a direction of future research with great potential would be to investigate other classes of methods that have been developed in the field, for example, wrapper, ensemble, and hybrid methods (Ang et al., 2016).

## Conclusion

Partitioning cancer patients based on RNA-seq data with the objective to identify subgroups is an important but also very challenging problem. The main difficulty is that only some genes are differentially expressed between the subgroups of interest and that several secondary factors affect gene expressions. Therefore, it is reasonable to assume that the clustering should be based on a set of carefully selected genes rather than all genes. The commonly used SD-approach, where genes with the highest SDs are selected, did not perform well in our study. We argue that SD is more likely to select genes affected by outliers, which in turn has a negative effect on the downstream cluster analysis. Although the performance in general is highly data-dependent, our study shows that selecting 1000 genes using the dip-test is a sensible selection approach, which performs considerably better than the SD-selection.

## Data Availability Statement

Publicly available datasets were analyzed in this study. This data can be found here: the gene expression data in this paper are available at https://gdac.broadinstitute.org/ under cohorts BRCA, LGG, KIRP, and STAD.

## Ethics Statement

Ethical review and approval was not required for the study on human participants in accordance with the local legislation and institutional requirements. The patients/participants provided their written informed consent to participate in this study.

## Author Contributions

DK was responsible for the data collection, pre-processing, and implementation of methods. All authors were involved in the evaluation, interpreting results, and preparing the final manuscript.

## Conflict of Interest

The authors declare that the research was conducted in the absence of any commercial or financial relationships that could be construed as a potential conflict of interest.

## Footnotes

1 https://gdac.broadinstitute.org
