# Supplementary material for: Comparison of Methods for Feature Selection in Clustering of High-Dimensional RNA-Sequencing Data to Identify Cancer Subtypes
Source: Front Genet. 2021 Feb 24;12:632620. doi: 10.3389/fgene.2021.632620 (PMC7943624; doi:10.3389/fgene.2021.632620)
Supplement: Supplementary file 1 [file Table_1.DOCX]

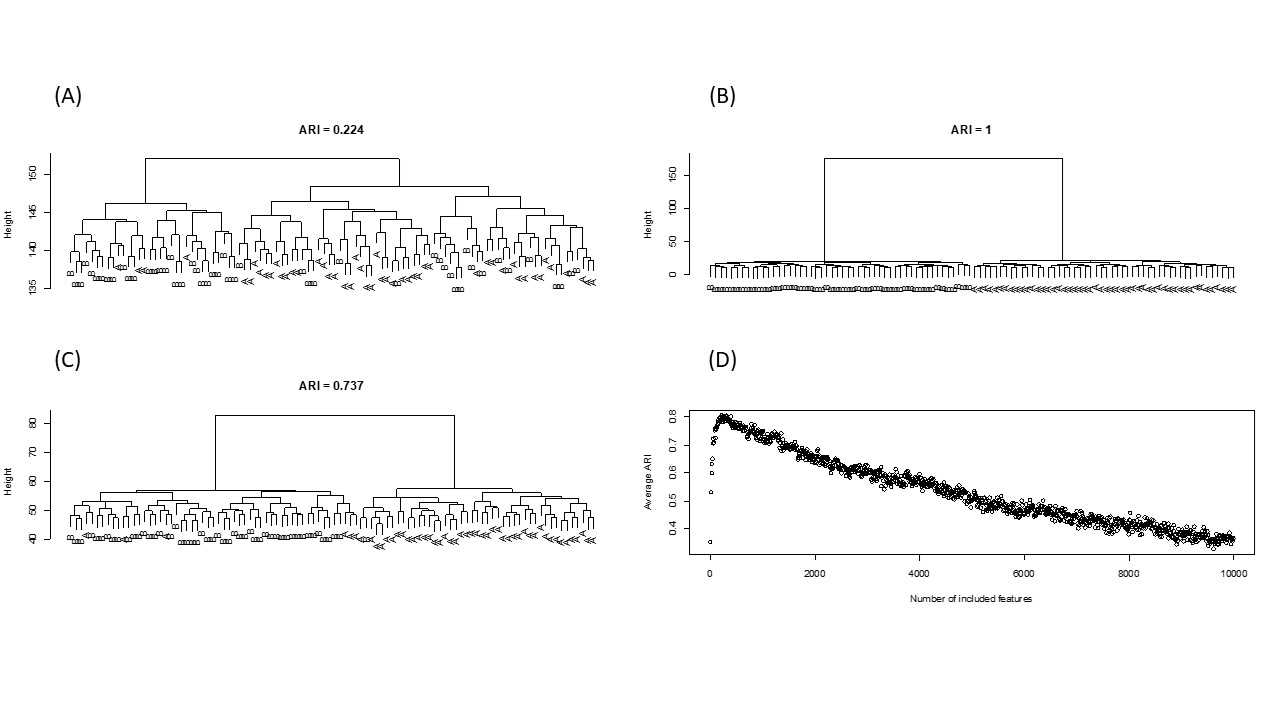


**Supplementary Figure 1.** Results from a simulation study with 100 informative features and 9900 non-informative features. Figures **A**-**C** show the adjusted Rand index for one realization using: all features (**A**), only informative features (**B**) and the 1000 features with the highest standard deviations (**C**). Figure **D** shows the average observed ARI (40 replicates) when the *k* features with the highest standard deviations were included, *k*=100, 200, … , 10000.
