# Supplementary material for: Comparison of Methods for Feature Selection in Clustering of High-Dimensional RNA-Sequencing Data to Identify Cancer Subtypes
Source: Front Genet. 2021 Feb 24;12:632620. doi: 10.3389/fgene.2021.632620 (PMC7943624; doi:10.3389/fgene.2021.632620)
Supplement: Supplementary file 3 [file Table_3.DOCX]

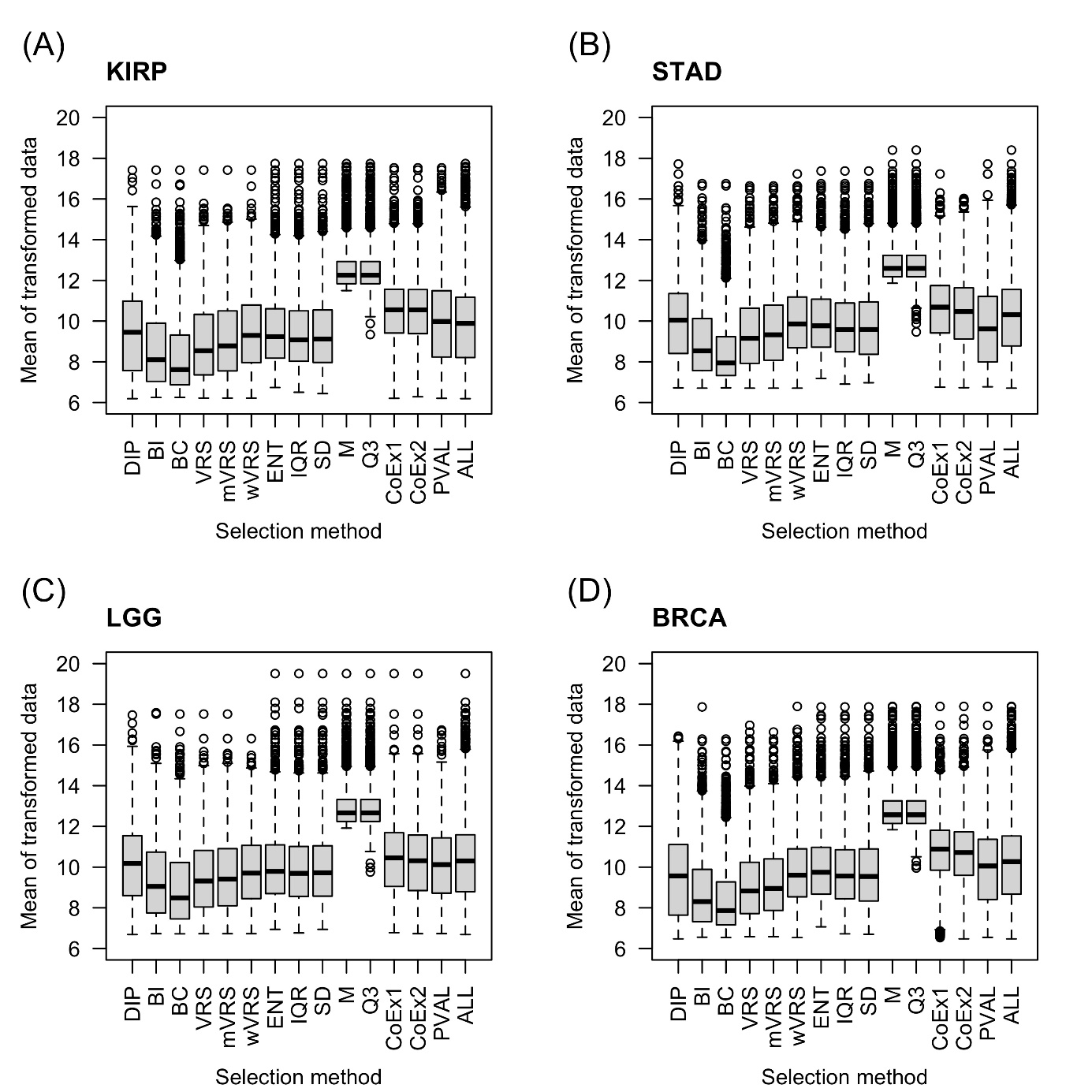


**Supplementary Figure 3.** Boxplots of mean expression values over all samples for 3000 selected genes. The figure shows the result for the data sets KIRP **(A)**, STAD **(B)**, LGG **(C)** and BRCA **(D)**. Each plot displays expression values of preprocessed data for the 13 gene selection methods, the positive control (PVAL) and the negative control (ALL) including all genes. The gene selection methods are: dip-test statistic (DIP), bimodality index (BI), bimodality coefficient (BC), variance reduction score (VRS), modified variance reduction score (mVRS), weighted variance reduction score (wVRS), entropy estimator (ENT), interquartile range (IQR), standard deviation (SD), mean value (M), third quartile (Q3), co-expression (CoEx1) and modified co-expression (CoEx2).
