# Supplementary material for: Comparison of Methods for Feature Selection in Clustering of High-Dimensional RNA-Sequencing Data to Identify Cancer Subtypes
Source: Front Genet. 2021 Feb 24;12:632620. doi: 10.3389/fgene.2021.632620 (PMC7943624; doi:10.3389/fgene.2021.632620)
Supplement: Supplementary file 6 [file Table_6.DOCX]

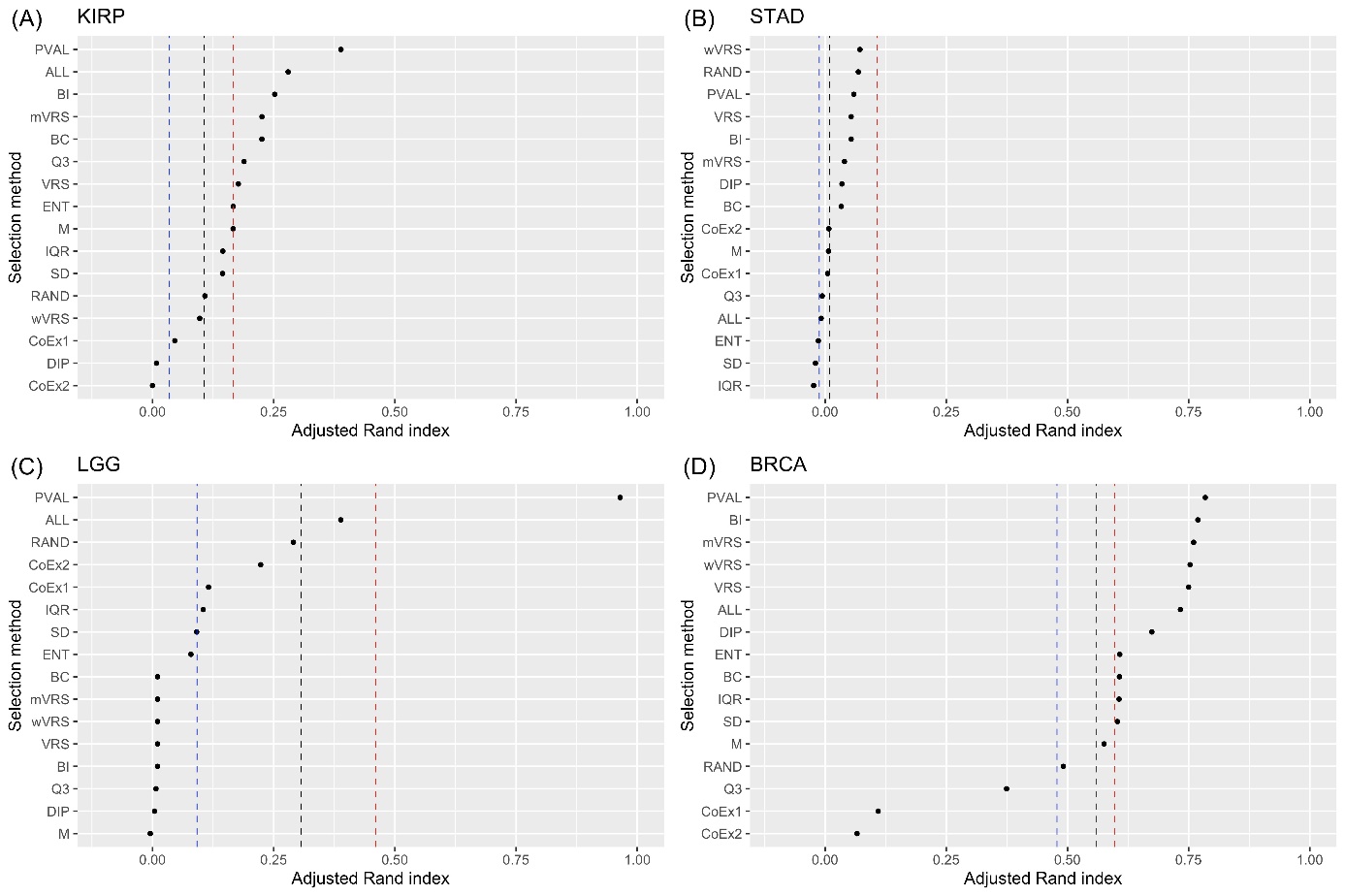


**Supplementary Figure 6.** Adjusted rand index for hierarchical clustering using the top 100 ranked genes on the datasets KIRP **(A)**, STAD **(B)**, LGG **(C)** and BRCA **(D)**. The vertical lines represent the first, second and third quartiles of a random selection. The feature selection methods on the y-axis are: dip-test statistic (DIP), bimodality index (BI), bimodality coefficient (BC), variance reduction score (VRS), modified variance reduction score (mVRS), weighted variance reduction score (wVRS), entropy estimator (ENT), interquartile range (IQR), standard deviation (SD), mean value (M), third quartile (Q3), co-expression (CoEx1), modified co-expression (CoEx2), the positive control (PVAL) and the two negative controls ALL and RAND. The performance of RAND is based on 1000 randomizations. The selection methods are ordered according to increasing performance.
