# Supplementary material for: Comparison of Methods for Feature Selection in Clustering of High-Dimensional RNA-Sequencing Data to Identify Cancer Subtypes
Source: Front Genet. 2021 Feb 24;12:632620. doi: 10.3389/fgene.2021.632620 (PMC7943624; doi:10.3389/fgene.2021.632620)
Supplement: Supplementary file 9 [file Table_9.DOCX]

|  | **DIP** | **BI** | **BC** | **VRS** | **mVRS** | **wVRS** | **ENT** | **IQR** | **SD** | **M** | **Q3** | **CoEx1** | **CoEx2** | **PVAL** | **RAND** |
| --- | --- | --- | --- | --- | --- | --- | --- | --- | --- | --- | --- | --- | --- | --- | --- |
| **KIRP100** | 0.01 | 0.21 | 0.23 | 0.21 | 0.21 | 0.23 | 0.17 | 0.25 | 0.20 | 0.06 | 0.12 | 0.02 | 0.00 | 0.29 | 0.13 |
| **STAD100** | 0.05 | 0.06 | 0.05 | 0.06 | 0.06 | 0.06 | -0.01 | 0.00 | 0.00 | 0.00 | -0.01 | 0.00 | 0.00 | 0.62 | 0.07 |
| **LGG100** | 0.01 | 0.01 | 0.01 | 0.01 | 0.01 | 0.01 | 0.09 | 0.07 | 0.08 | 0.00 | 0.01 | 0.39 | 0.29 | 0.95 | 0.30 |
| **BRCA100** | 0.75 | 0.77 | 0.67 | 0.76 | 0.76 | 0.77 | 0.69 | 0.76 | 0.74 | 0.13 | 0.10 | 0.10 | 0.06 | 0.78 | 0.46 |
| **KIRP1000** | 0.20 | 0.23 | 0.20 | 0.25 | 0.24 | 0.24 | 0.20 | 0.23 | 0.20 | 0.25 | 0.34 | 0.05 | 0.03 | 0.32 | 0.25 |
| **STAD1000** | 0.46 | 0.45 | 0.42 | 0.28 | 0.28 | 0.26 | 0.07 | 0.07 | 0.07 | 0.00 | 0.01 | 0.00 | 0.00 | 0.56 | 0.03 |
| **LGG1000** | 0.60 | 0.12 | 0.11 | 0.12 | 0.12 | 0.12 | 0.11 | 0.11 | 0.11 | 0.54 | 0.45 | 0.34 | 0.16 | 0.83 | 0.33 |
| **BRCA1000** | 0.71 | 0.73 | 0.71 | 0.73 | 0.75 | 0.75 | 0.73 | 0.72 | 0.67 | 0.72 | 0.72 | 0.08 | 0.06 | 0.77 | 0.70 |
| **KIRP3000** | 0.29 | 0.23 | 0.24 | 0.24 | 0.31 | 0.29 | 0.31 | 0.32 | 0.31 | 0.19 | 0.19 | 0.04 | 0.02 | 0.29 | 0.27 |
| **STAD3000** | 0.46 | 0.23 | 0.15 | 0.37 | 0.37 | 0.04 | 0.12 | 0.12 | 0.12 | 0.00 | 0.00 | 0.00 | 0.00 | 0.60 | 0.00 |
| **LGG3000** | 0.55 | 0.12 | 0.12 | 0.12 | 0.12 | 0.13 | 0.19 | 0.19 | 0.19 | 0.49 | 0.41 | 0.18 | 0.18 | 0.72 | 0.33 |
| **BRCA3000** | 0.72 | 0.70 | 0.72 | 0.72 | 0.74 | 0.74 | 0.69 | 0.72 | 0.68 | 0.70 | 0.73 | 0.09 | 0.08 | 0.75 | 0.71 |

Supplementary Table 2. Adjusted Rand index for 13 feature selection methods, a negative (RAND) and positive control (PVAL) for data sets KIRP, STAD, LGG and BRCA. The performance is based on k-means clustering (k=2) clustering. The table shows results for selection of top ranked genes at three levels: 100, 1000 and 3000 genes. Adjusted Rand index when including all genes was obtained as 0.28, -0.01, 0.39 and 0.73 for KIRP, STAD, LGG and BRCA respectively. The gene selection methods are dip-test statistic (DIP), bimodality index (BI), bimodality coefficient (BC), variance reduction score (VRS), modified variance reduction score (mVRS), weighted variance reduction score (wVRS), entropy estimator (ENT), interquartile range (IQR), standard deviation (SD), mean value (M), third quartile (Q3), co-expression (CoEx1) and modified co-expression (CoEx2).
