# Supplementary material for: Comparison of Methods for Feature Selection in Clustering of High-Dimensional RNA-Sequencing Data to Identify Cancer Subtypes
Source: Front Genet. 2021 Feb 24;12:632620. doi: 10.3389/fgene.2021.632620 (PMC7943624; doi:10.3389/fgene.2021.632620)
Supplement: Supplementary file 11 [file Table_11.docx]

Supplementary Table 4.

Rank of feature selection methods for data sets KIRP, STAD, LGG and BRCA based on adjusted Rand index. The performance is based on k-means clustering (k=2). The table shows results for selection of top ranked genes at three levels: 100, 1000 and 3000 genes. The gene selection methods are: dip-test statistic (DIP), bimodality index (BI), bimodality coefficient (BC), variance reduction score (VRS), modified variance reduction score (mVRS), weighted variance reduction score (wVRS), entropy estimator (ENT), interquartile range (IQR), standard deviation (SD), mean value (M), third quartile (Q3), co-expression (CoEx1), modified co-expression (CoEx2), the positive (PVAL) and negative control (RAND).

|  | **DIP** | **BI** | **BC** | **VRS** | **mVRS** | **wVRS** | **ENT** | **IQR** | **SD** | **M** | **Q3** | **CoEx1** | **CoEx2** | **PVAL** | **RAND** |
| --- | --- | --- | --- | --- | --- | --- | --- | --- | --- | --- | --- | --- | --- | --- | --- |
| **KIRP100** | 14 | 6 | 3.5 | 6 | 6 | 3.5 | 9 | 2 | 8 | 12 | 11 | 13 | 15 | 1 | 10 |
| **STAD100** | 7 | 4.5 | 8 | 4.5 | 4.5 | 4.5 | 13 | 13 | 13 | 10 | 15 | 9 | 11 | 1 | 2 |
| **LGG100** | 13 | 10 | 14 | 10 | 10 | 10 | 5 | 7 | 6 | 15 | 10 | 2 | 4 | 1 | 3 |
| **BRCA100** | 7 | 2 | 10 | 5.5 | 4 | 3 | 9 | 5.5 | 8 | 12 | 14 | 13 | 15 | 1 | 11 |
| **KIRP1000** | 10.5 | 8 | 10.5 | 4.5 | 6.5 | 6.5 | 12.5 | 9 | 12.5 | 4.5 | 1 | 14 | 15 | 2 | 3 |
| **STAD1000** | 2 | 3 | 4 | 5.5 | 5.5 | 7 | 8.5 | 8.5 | 10 | 14.5 | 12 | 14.5 | 13 | 1 | 11 |
| **LGG1000** | 2 | 9.5 | 14 | 9.5 | 9.5 | 9.5 | 12.5 | 12.5 | 15 | 3 | 4 | 5 | 7 | 1 | 6 |
| **BRCA1000** | 11 | 5 | 10 | 4 | 3 | 2 | 6 | 8 | 13 | 9 | 7 | 14 | 15 | 1 | 12 |
| **KIRP3000** | 6 | 11 | 9.5 | 9.5 | 3 | 6 | 3 | 1 | 3 | 12.5 | 12.5 | 14 | 15 | 6 | 8 |
| **STAD3000** | 2 | 5 | 6 | 3.5 | 3.5 | 10 | 8 | 8 | 8 | 12.5 | 12.5 | 11 | 14 | 1 | 15 |
| **LGG3000** | 2 | 14.5 | 14.5 | 12.5 | 12.5 | 11 | 7 | 7 | 7 | 3 | 4 | 9.5 | 9.5 | 1 | 5 |
| **BRCA3000** | 7 | 11 | 7 | 5 | 2.5 | 2.5 | 12 | 7 | 13 | 10 | 4 | 14 | 15 | 1 | 9 |
| **Mean rank** | **7.0** | **7.5** | **9.3** | **6.7** | **5.9** | **6.3** | **8.8** | **7.4** | **9.7** | **9.8** | **8.9** | **11.1** | **12.4** | **1.5** | **7.9** |
