# Supplementary material for: Comparison of Methods for Feature Selection in Clustering of High-Dimensional RNA-Sequencing Data to Identify Cancer Subtypes
Source: Front Genet. 2021 Feb 24;12:632620. doi: 10.3389/fgene.2021.632620 (PMC7943624; doi:10.3389/fgene.2021.632620)
Supplement: Supplementary file 12 [file Table_12.DOCX]

|  |  | 25 % | | | 50 % | | |
| --- | --- | --- | --- | --- | --- | --- | --- |
|  |  | **BI** | **mVRS** | **SD** | **BI** | **mVRS** | **SD** |
| KIRP | **DIP** | 0.00 | 0.00 | 0.01 | 0.00 | 0.01 | 0.04*** |
|  | **BI** |  | 0.00 | 0.01 |  | 0.01 | 0.03*** |
|  | **mVRS** |  |  | 0.01* |  |  | 0.04*** |
| STAD | **DIP** | -0.02 | -0.02 | 0.06*** | 0.00 | 0.00 | 0.02 |
|  | **BI** |  | 0.00 | 0.07*** |  | 0.00 | 0.02* |
|  | **mVRS** |  |  | 0.08*** |  |  | 0.02 |
| LGG | **DIP** | 0.08*** | 0.06*** | 0.04* | 0.08*** | 0.08*** | 0.09*** |
|  | **BI** |  | -0.02 | -0.04** |  | 0.00 | 0.01 |
|  | **mVRS** |  |  | -0.02 |  |  | 0.01 |
| BRCA | **DIP** | 0.02*** | 0.01 | 0.03*** | 0.02*** | 0.01 | 0.03*** |
|  | **BI** |  | -0.01 | 0.01** |  | -0.02** | 0.01 |
|  | **mVRS** |  |  | 0.03*** |  |  | 0.03*** |

**Supplementary Table 5.** The mean value of the *smallest fraction*-differences (row method – column method) when different pairs of variable selection methods (the dip-test (DIP), bimodality index (BI), modified variance reduction score (mVRS) and standard deviation (SD)) were compared using 100 simulated data sets which each contained 100 samples. The smallest fraction is the percentage of individuals observed in the smallest group for a bivariat clustering. Simulations were made for the data sets KIRP, STAD, LGG and BRCA, and two types of data sets were simulated: unbalanced data where 25 % of the individuals belonged to the minor class and a balanced data set were 50 % of the individuals belonged to each of the two classes. The one sample t-test was used to test if the mean difference deviated from zero. Positive (negative) differences indicate that the row-method had more (less) observations in the minor group than the column method. Here *, ** and *** denote a significant result at the 0.05, 0.01 and 0.001 significance level respectively.
